# Treatment management algorithm for natural frozen embryo transfer cycles using a real-time ovulation prediction machine learning model

**DOI:** 10.1038/s41598-026-42921-1

**Published:** 2026-03-08

**Authors:** Eden Moran, Ariel Hourvitz, Almog Luz, Nevo Itzhak, Rohi Hourvitz, Michal Youngster, Micha Baum, Ettie Maman

**Affiliations:** 1 FertilAI, Ramat Gan, Israel; 2 Shamir Medical Center, In Vitro Fertilization Unit, Department of Obstetrics and Gynecology, Zerifin Israel, Faculty of Medical & Health Sciences, Tel Aviv University, Tel Aviv, Israel; 3 Sheba Medical Center In Vitro Fertilization Unit, Department of Obstetrics and Gynecology, Ramat Gan, Israel; 4 Faculty of Medical & Health Sciences, Tel Aviv University, Tel-Aviv, Israel; 5 Herzliya Medical Center, In Vitro Fertilization Unit, Herzliya, Israel

**Keywords:** Artificial intelligence, Machine learning, Ovulation determination, Natural cycle, Frozen embryo transfer, Software, Infertility

## Abstract

Our aim was to develop an AI-based NC-FET Treatment Management Algorithm (NTMA), decision-support system that predicts ovulation in real time to manage and optimize natural frozen embryo transfer cycle (NC-FET) scheduling. The algorithm was developed using a “teacher-student” machine learning approach and was trained on a total of 3,975 labeled NC-FET, including 3,432 training cycles and 543 test cycles. A second test group included 166 documented ovulation cycles (documented follicular rupture and LH surge in two consecutive days of ultrasound scans). The algorithm showed high ovulation detection accuracy in both tests’ groups particularly one day before and the day of ovulation (95.4% and 94.6% in the labeled test group and 95.5% and 95.3% in the documented test group, respectively). Most influential predictive features of the algorithm included LH levels, the estrogen/progesterone ratio, and leading follicle size during the monitored test days. The NTMA yielded 92.04% correct prediction 7 in identifying ovulation with an average of 3.1 tests per cycle. We propose an AI-based algorithm for the complete management of a NC-FET. The algorithm shows high accuracy in predicting the time of ovulation and therefore can serve as a useful decision support tool for clinicians in their daily practice. Prospective studies are warranted to validate these results.

**Supplementary Information:**

The online version contains supplementary material available at 10.1038/s41598-026-42921-1.

## Introduction

 The introduction of vitrification and advancements in cryopreservation techniques have led to higher embryo survival rates and, consequently, more available embryos^1^. As a result, the number of frozen embryo transfer (FET) cycles has increased in recent years^2^. Among many fertility physicians, one of the preferred methods is time-frozen embryo transfer based on the natural cycle (NC-FET)^3,4^. NC-FET is associated with lower rates of early pregnancy loss^5,6^ and reduced obstetric complications, such as hypertensive disorders of pregnancy and large-for-gestational-age infants, compared to cycles involving artificial endometrial preparation^4,7,8^. Additionally, a recent systematic review and meta-analysis by Wu et al.^9^ reported increased live birth rates (LBRs) in NC-FET cycles compared to artificial FET cycles.

NC-FET requires close monitoring of the natural menstrual cycle to identify the timing of ovulation. Currently, the method considered by many as the most accurate method in NC-FET is the use of a combination of blood tests (Estrogen, Progesterone, and LH) and ultrasound monitoring of the growth and rupture of the leading follicle during ovulation^10–12^. This method requires patients to undergo several follow-up tests throughout the month to accurately monitor ovulation and consequently requires extensive expertise and time on the part of the physician.

In recent years, artificial intelligence (AI) technologies have been introduced in the field of reproduction, offering a range of benefits that could revolutionize the way we approach fertility treatments^13^. Several recently published studies utilized machine learning technologies in the field of reproduction including predicting IVF outcomes^14^, optimizing ovarian stimulation^15,16^, sperm selection^17^, trigger day^18–20^, IUI (Intrauterine Insemination) scheduling^21,22^, workload balancing^23^ embryo selection^24^ and euploidy prediction^25^. In a recent study, we examined the accuracy of AI-based ovulation prediction model on the results of NC-FET cycles. We showed that the pregnancy rate in NC-FET cycles, where the transfer was performed according to the ovulation date predicted by the model (matched cycles), was 9% higher than in cycles without such a match between the model and the physician’s ovulation time determination^26^.

Based on the model developed in a previous study, we introduce in this study a novel decision-support AI software designed to predict ovulation in real-time and fully manage the treatment of a natural frozen embryo transfer cycle. The algorithm supports the treatment cycle from its early stages, schedules the necessary tests, and ultimately predicts ovulation, thereby determining the optimal date for embryo transfer.

## Results

In this section, we first present the performance of the real-time ovulation day prediction model used by the NC-FET Treatment Management Algorithm (NTMA), followed by the evaluation of the NTMA itself. As detailed in the Materials and Methods section, the real-time prediction model was trained on data labeled according to a previously published ovulation prediction model^26^, using the “student-teacher” approach^27^. The real-time ovulation day prediction model was trained on instances comprising one or two test days, with features derived from these days.

### Model performance

**Fig. 1:**
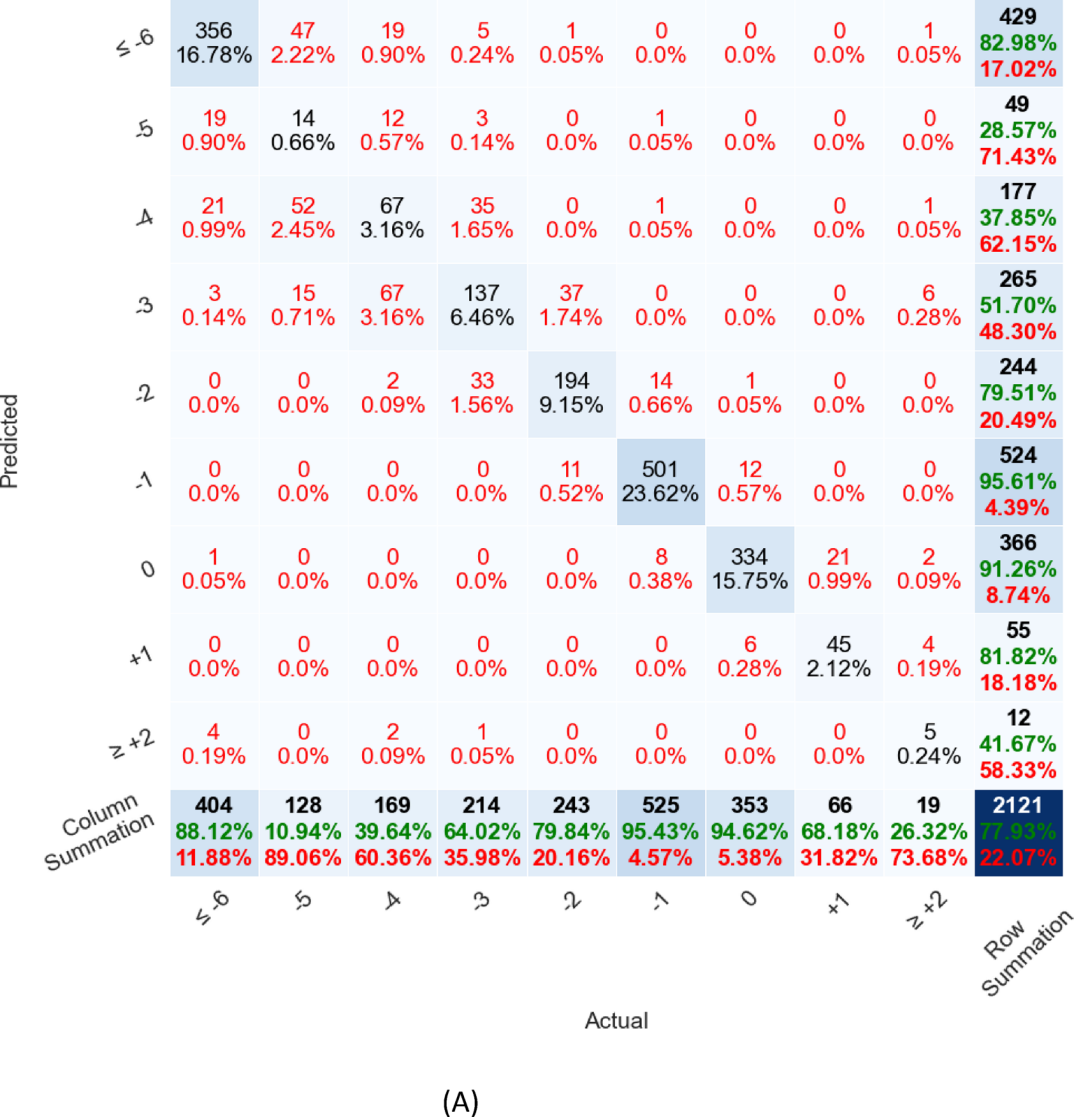

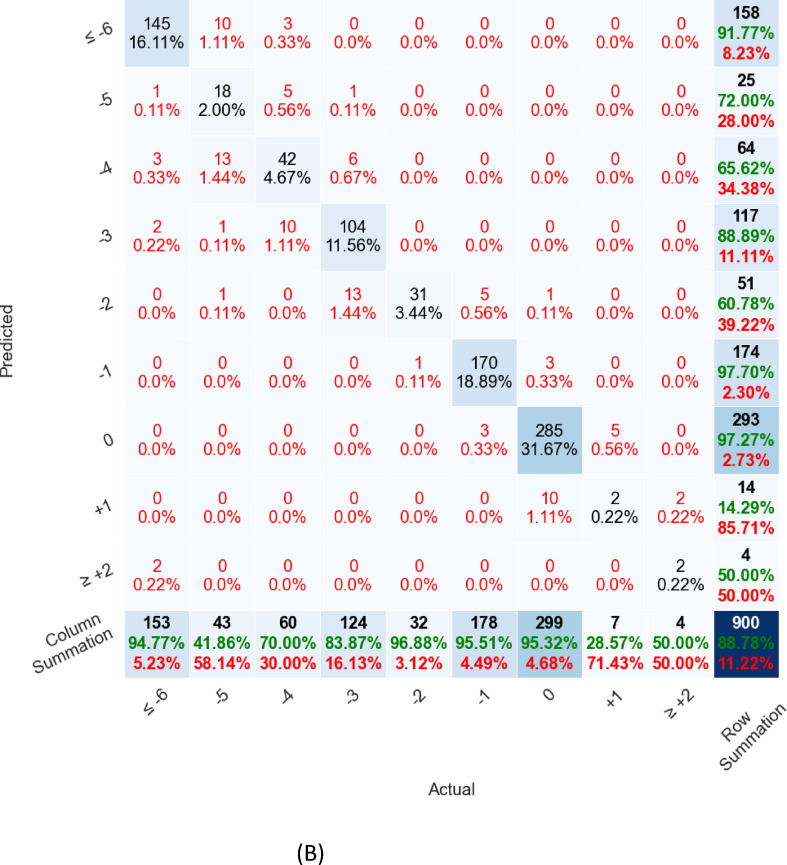
(**A**) Model performance on the labeled dataset displayed as a confusion matrix, (**B**) Model performance on the documented ovulation dataset

The real-time ovulation day prediction model’s performance on each test set is represented as a confusion matrix (actual vs. predicted relative-to-ovulation days). Figure 1A shows the results on the Labeled Dataset, and Fig. 1B presents the results on instances on the Documented Ovulation Dataset. In those figures, the model shows strong predictive power in two key ranges: far from ovulation (≤ -6) and close to ovulation (-1 or 0). For the far-from-ovulation predictions (≤ -6), we observed improved model performances because the test days are early enough to capture patterns where ovulation is not imminent, allowing for more accurate predictions that align with these distant days. On the other hand, the model also performs well around − 1 and 0, as these ranges correspond to days immediately before or on the ovulation day itself. During these days, the biological signals, such as hormonal changes, are most prominent, providing the model with the information needed to make accurate predictions. As a result, the combination of early test days for distant predictions and clear hormonal patterns near ovulation leads to strong performance across these ranges.

Figure 1A and B: Model performance. Columns represent instances’ actual classes, relative to the instance’s latest test day. Rows represent the model-predicted classes. The number and percentage of total occurrences for actual vs. prediction pairs are presented in each cell. Percentages were normalized cumulative sum of 100%. The bottom row and right column highlight the accuracy in green and the errors in red, respectively. The number of occurrences is positioned at the top of each cell. The bottom-right cell signifies the overall model accuracy. In Figs. 1 A and B, the model shows strong predictive power in two key ranges: far from ovulation (≤ -6) and close to ovulation (-1 or 0).

### Feature importance

**Fig. 2:**
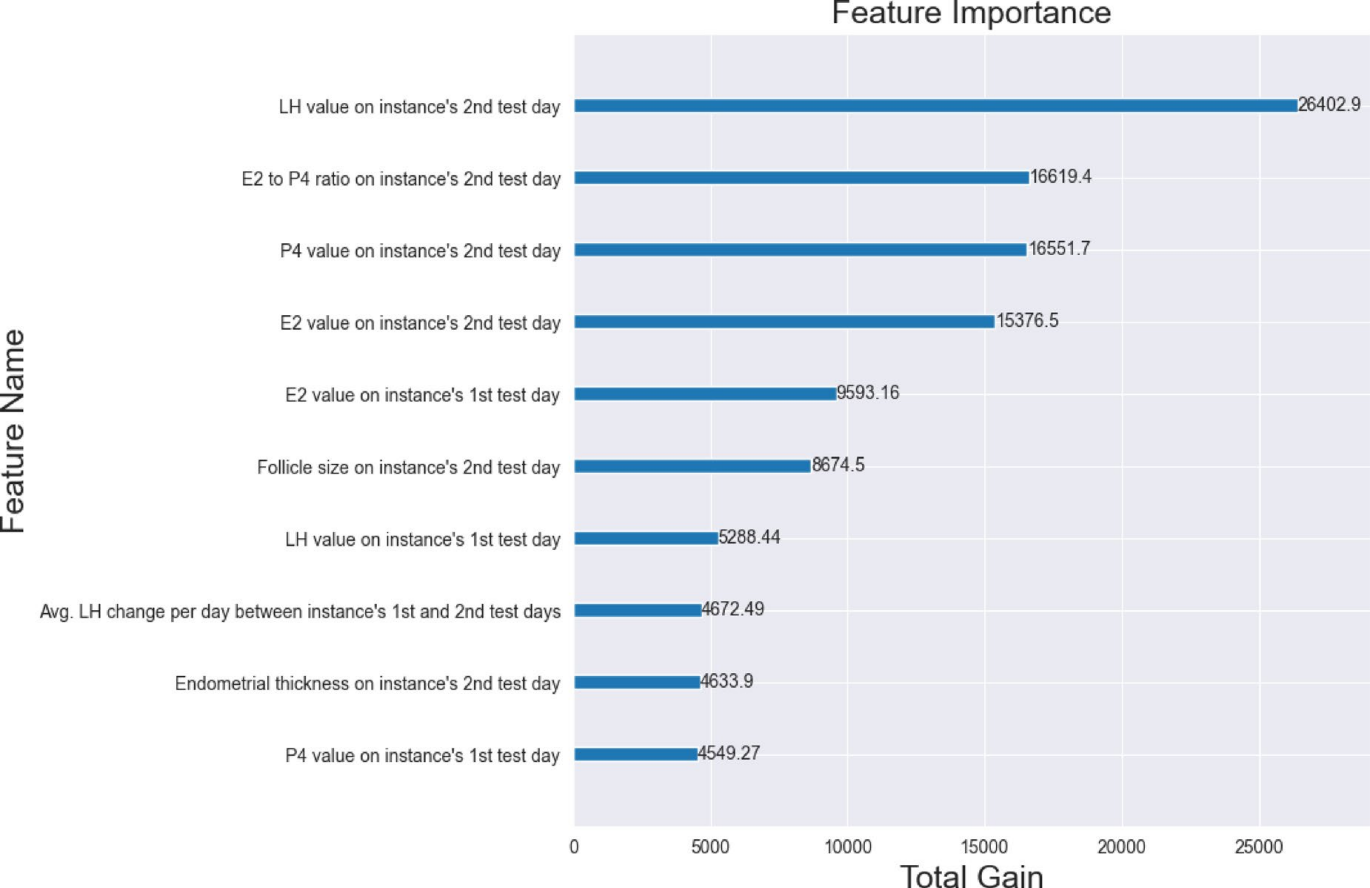
Real-time ovulation day prediction model’s most influential features. For each feature, the relative contribution is presented as the total gain. Summary of the real-time ovulation day prediction model’s ten most important features by total gain of each feature.

The top ten influential features, used by the model to determine ovulation day, are presented in Fig. 2. The terms “first” and “second” test days refer to their chronological order within each instance. In cases where only a single test day is used, it is considered both the first and second day. Overall, features associated with the second test day showed greater influence than those linked to the first test day. The most influential features included LH, the ratio between estrogen (E2) and progesterone (P4), P4, and E2 on the instance’s second test day. The other features used by the model were: E2 value on the instance’s first test day, leading follicle size on the instance’s second test day, LH value on the instance’s first test day, average LH difference per day between the instance’s first and second test days, endometrial thickness value on the instance’s second test day, and E2 value on the instance’s first test day. The LH value on the second test day had higher importance compared to the LH value on the first test day. This is consistent with the physiology of the peri-ovulatory LH rise. Because the second test day is typically closer to ovulation, the model naturally assigns it greater weight. In contrast, the LH value on the first test day contributes less, as it is usually obtained earlier in the follicular phase, when LH levels are lower, more stable, and therefore less informative.

### NTMA performance

Table 1 shows the results for NTMA performance. The algorithm’s performance was estimated using a statistical simulation-based approach that incorporates cycle-day ovulation probabilities, model prediction accuracy, and treatment-management logic (see Supplementary Part 3: NTMA’s Performance Evaluation). The following metrics were used to evaluate performance.:

- Correct Prediction: The algorithm correctly determined the ovulation day.
- Incorrect Prediction: The algorithm, based on the prediction of the real-time ovulation prediction model, was not able to correctly identify the ovulation day.
- No Prediction: The algorithm could not accurately predict ovulation as the test was performed two or more days following ovulation.
- Number of tests performed.

Overall, the model demonstrates a 92.04% correct prediction rate, with a 1.16% no prediction rate (NTMA’s Objective 1), and incorrect prediction rate of 6.79%. An average number of tests of 3.10 were performed. The NTMA approach reduces the number of blood tests and ultrasounds required. Table 1 highlights that the average number of tests using NTMA is 3.10, compared to an average of 3.54 [3.32–3.67] blood tests (excluding baseline tests) in the Labeled Dataset and average of 3.48 [3.35–3.61] number of ultrasounds in the Labeled Dataset (excluding baseline tests). Comparison of monitoring burden (number of blood tests and ultrasounds) is reported only for the Labeled Dataset. Specifically, the Documented Ovulation Dataset was created from cycles with two consecutive days of testing on the day before and the day of ovulation and was used solely for model validation; therefore, it does not represent routine clinical monitoring practices.

**Table 1:** Summary results of the NTMA.

Correct prediction rate (%)	92.04 [91.75–92.34]
Incorrect prediction rate (%)	6.79% [6.53–7.05]
No prediction rate (%)	1.16 [0.98–1.33]
Average number of tests	3.10 [3.09–3.11]

[ ] 95% Confidence Interval.

## Discussion

Embryo transfer in the natural cycle is currently considered the preferred method for frozen embryo transfer^3,4^. In this novel study, we present an AI-based NC-FET treatment management algorithm that uses an ovulation determination model to schedule embryo transfer time. With an average of 3.1 follow-up test days per cycle, the model successfully determined the ovulation date in 92.04% of cases. This new algorithm can fully manage frozen cycles and serve as a decision support system for NC-FET.

In the previous article^26^, we demonstrated that, theoretically, pregnancy rates could be improved when the ovulation date was matched with the model’s recommendation. In this article, however, we present an algorithm trained on the results of the previous model, capable of achieving the same level of prediction accuracy while completely managing treatment. The real time ovulation prediction algorithm was developed using the teacher-student method^27^. The ovulation labeling model (teacher) was trained on hundreds of cycles in which ovulation was determined by fertility experts. Afterward, the real-time (student) algorithm was trained on several thousand cycles where the ovulation date was labeled by the ovulation labeling model. This approach allows models to be trained on a large number of treatment cycles without the need for manual labeling. However, because the real time algorithm was trained on labels generated by the teacher model rather than directly on expert annotations for all cycles, any residual inaccuracies of the teacher may also be reflected in the student. This reliance on model generated labels should be considered when interpreting the results. Developing a treatment management algorithm that attempts to optimize the treatment by selecting the optimal test days using retrospective data proved challenging. The test days in the data were selected by the attending physicians and rarely matched the algorithm’s recommendation; therefore, the algorithm performance could not be tested directly using the available treatment cycles and the statistical method described in the materials and methods was used. This method has the advantage of being capable of analyzing the performance of the treatment management algorithm just by using statistical information about the probability of a patient to ovulate on each cycle day, the different error probabilities of the ovulation determination model and the logic of the treatment management algorithm. The method allowed us to quickly repeat through multiple versions of the treatment management algorithm logic and select the optimal one.

The accuracy of ovulation determination by the model was tested using two datasets: the ovulation labeling model-Labeled Dataset and the Documented Ovulation Dataset. The advantages of the model-Labeled Dataset include its larger size and random selection. In contrast, the Documented Ovulation Dataset is smaller, however ovulation there was confirmed using two “gold standard” criteria: evidence of follicular rupture and an LH surge^3,4,28,29^. It is important to note that there is no standard definition for an LH surge; to address this, we define an LH surge as LH > 17 IU/L^30^ with a documented decrease in LH level the following day. The fact that accuracy of ovulation prediction model in the Labeled Dataset was similar to that in a documented ovulation test set (up to 95.4 and up to 95.5%, respectively) strongly suggests that the algorithm is highly accurate in determining the true ovulation time. Nevertheless, additional prospective studies are needed to validate the model’s impact on NC-FET timing and clinical outcomes.

The development of this algorithm raises some clinical questions. One might question the necessity of a natural ovulation detection model when ovulation can be timed through the administration of an hCG trigger and a schedule ovulation and embryos transfer (modified NC-FET). While the question of whether frozen embryo transfer should occur in a fully natural cycle versus a modified natural cycle lies outside the primary focus of this article, it remains without a definitive answer. Some studies indicate no significant difference in success rates between modified cycles and natural cycles, which generally necessitate more frequent clinic visits and monitoring^3,31,32^. Conversely, other studies suggest an advantage for the fully natural cycle approach^33–35^. Variability in reported success rates may be attributed to factors such as the precise timing of hCG administration, which, if misaligned, can impair endometrial receptivity or lead to suboptimal corpus luteum function. Future approaches may benefit from a hybrid model that uses the strengths of both strategies—preserving the natural cycle where feasible, while also providing the option to control ovulation timing through scheduled hCG administration when needed. The most effective pathway to achieve this balance likely lies in the development of AI-driven algorithms. Such models could integrate serial hormonal measurements, ultrasound findings and scheduling constraints to propose individualized treatment strategies. In particular, they could recommend hCG triggering when the predicted transfer day based on spontaneous ovulation is not feasible due to patient or clinic constraints.

The algorithm presented in this article is designed to identify ovulation, and thereafter the physician can determine embryo transfer time according to embryo age, making it suitable for all types of frozen embryos. It is important to emphasize that the proposed NTMA is specifically designed for frozen embryo transfer cycles. This includes that if the ovulation date identified by the algorithm has passed by one day, frozen embryos can still be transferred.

As expected, LH, estrogen and progesterone were the major factors used by the model for ovulation prediction. Interestingly, one of the influential features affecting the algorithm performance was the Estrogen/Progesterone ratio on the prediction day. The finding of the Estrogen/Progesterone ratio as an influential factor is in agreement with previous studies^33,34^ that consider the decrease in the ratio of Estrogen/Progesterone metabolites in urine to correspond to the day of luteal transition in ovulatory women. It is important to note that the Estrogen/Progesterone ratio is not in routine daily clinical use, probably because of its relative complexity. However, the ability of AI algorithms to quickly process data, increases the possibility of using this feature and many others, such as the ratios and derivative between different hormones in consecutive tests. As can be seen in Fig. 1, the model uses these additional features to increase the accuracy of ovulation determination and optimal FET timing.

While this retrospective study highlights the NTMA’s clinical utility, prospective validation is needed to assess its impact on pregnancy outcomes. The study does not include pregnancy rates, which is essential to fully assess the algorithm’s clinical utility and effectiveness in improving FET success. However, the algorithm was trained on labeled data, and the labeling model was retrospectively tested on NC-FET cycles, showing a 9% increase in pregnancy rates in cycles where ovulation matched the model’s prediction^26^. Another drawback of this study is that AI algorithms are susceptible to bias, which can arise from data selection or labeling processes. Although the algorithm was tested using two datasets to minimize this risk, some bias may still persist.

In conclusion, to our knowledge, this is the first highly accurate and useful AI-based algorithm designed for the full management of an IVF protocol. The performance achieved by the NTMA model for ovulation detection and management of NC-FET cycles are reassuring. The high accuracy is attributed to the capability of the algorithm to analyze effectively and accurately multiple and complex features. We believe that this algorithm and others that will follow can serve clinicians in their daily practice, improving clinical outcomes while increasing their clinical activity. We anticipate the growing availability of this and other AI algorithms will greatly impact the accessibility, cost and success of fertility treatments.

## Materials and methods

The study was approved by the Institutional Review Board of Herzliya Medical Center (HMC-0008-21). Due to the retrospective nature of the study, informed consent was waived by the Institutional Review Board of Herzliya Medical Center. We confirm that the study was preformed in accordance with relevant guidelines and regulations.

### The “Student-Teacher” approach

The Ovulation Labeling Model (“Teacher”): The teacher model that was previously described^26^, was trained and evaluated on NC-FET cycles between 2018 and 2023. Briefly, the model was first trained on 399 cycles, where ovulation timing was determined by a majority decision from three reproductive endocrinology and infertility independent specialists (REI). The model was evaluated on 101 cycles where ovulation was determined by the same majority method and 101 cycles with documented ovulation. The model achieved high accuracy in these test groups, 93.85% and 92.89% matching rates with the REI and Documented Ovulation Datasets, respectively. We used this *“Teacher”* prediction on multiple instances from a cycle to determine the ovulation day, relying on aggregated information from the cycle’s data, in a retrospective way, for a final decision (see Supplementary Material Part 1).

The Real-Time Ovulation Day Prediction Model (“Student”): The real-time ovulation day prediction model stands at the core of the treatment management algorithm and is used to predict the probability of a patient ovulating. The real-time ovulation day prediction model was trained using a “student-teacher” approach^27^. The teacher algorithm was used to label the precise ovulation day in NC-FET cycles, which were then used to train the real-time ovulation day prediction model (“student”) accurately. Achieving an accurate real-time model required extensive training on a large dataset, which would not have been feasible with only a few hundred manually labeled cycles by fertility experts, as was done with the teacher model. The primary distinction between the ovulation labeling model and the real-time ovulation day prediction model lies in how they are applied. In contrast to the teacher model, the real-time ovulation day prediction model is optimized for real-time prediction, considering only the latest instance to provide immediate, actionable insights without the context of the entire cycle. This approach enabled the student model to accurately predict ovulation several days in advance, unlike the ovulation labeling model (teacher model).

Natural FET Treatment Management Algorithm (NTMA): In this study, we propose a treatment management algorithm for spontaneous natural FET cycles that fully manages the cycle. After the Real-Time Ovulation Day Prediction Model predicts the day relative to ovulation, a set of rules (the NTMA) is applied explained further below. At first, the NTMA recommends when the patient should come for the initial test day. Then, after each test day, the algorithm considers the patient’s likelihood and timing of ovulation, provided by the machine learning model, and recommends if and when another test day should be performed, or if an accurate prediction of ovulation can be made. Further details regarding the logic of the NTMA are provided in the Methods section.

### Datasets

#### Labeled dataset

To train and evaluate the real-time ovulation day prediction model (“Student”), we used the ovulation day determination model (“Teacher”) to generate the dataset. The initial dataset is from Herzliya Medical Center, Israel, and consisted of 5,162 natural FET cycles performed between August 2018 and May 2024. Filtering out cycles with uncertain class prediction (i.e., the model could not accurately predict when ovulation will occur given the existing test results) of the teacher algorithm^26^ reduced the number to 4,361 cycles. Removing cycles with expert prediction, utilized in the teaching process of the teacher algorithm, resulted in the remaining 3,975 cycles.

After the teacher model completed ovulation determination on the cycles, we used the resulting dataset to train and evaluate the real-time ovulation day prediction model (“Student”). The data obtained for each cycle consisted of baseline characteristics, including the patient’s age at the time of embryo freezing and embryo transfer, body mass index (BMI), and cycle characteristics, including the leading follicle’s size, endometrial thickness, blood test results for estradiol, progesterone, and LH levels, and day of embryo transfer. Table 2 shows patients’ characteristics of the real-time ovulation day prediction model training and test cycles. The table presents baseline patient characteristics only. Time-dependent variables, such as hormone levels and embryo transfer day, were not included because they vary across test days and are analyzed dynamically within the model.

**Table 2:** Baseline characteristics of patients included in the labeled dataset.

Number of cycles	Training sets*	Test set	*P*-value
	3,432	543	....
Mean patient age [y]** (Mean ± SD)	36.44 ± 5.80	36.33 ± 5.93	0.58
BMI [kg/m2] (Mean ± SD)	24.27 ± 5.66	24.68 ± 5.51	0.05
Smokers (%)	7.66	7.37	0.65
Ovulation day as marked by as Ovulation Labeling Model (Mean ± SD)	16.00 ± 5.08	16.34 ± 4.95	0.05
Endometrial width [mm]*** (Mean ± SD)	9.55 ± 1.99	9.59 ± 2.14	0.95

Presented as mean ± standard deviation (SD).

*Including the train and validation sets that were used to learn the model.

**At the time of embryo transfer.

***As measured on the last test day.

#### Documented ovulation dataset

The Documented Ovulation Dataset included 166 cycles from 2019 to 2024 and was used only to evaluate the real-time ovulation day prediction model performance (a test set only). Inclusion criteria were: two consecutive days of blood and ultrasound tests, documented follicular rupture of a single primary follicle measuring ≥ 17 mm, and documented LH surge, defined as LH ≥ 17 IU on the initial day^30^, followed by a decrease on the next day. The LH cut-off of 17 IU/L was used solely for defining ovulation in the Documented Ovulation Dataset and does not affect model training or feature-importance estimation. The student model uses continuous LH values rather than any threshold.

Table 3 shows patients’ characteristics of the training cycles of the Labeled Dataset compared with the Documented Ovulation Dataset.

**Table 3:** Baseline characteristics of patients included in the labeled dataset compared with the documented ovulation dataset.

Number of cycles	Training sets*	Documented ovulation set	*P*-value
	3,432	166	
Mean patient age [y]** (Mean ± SD)	36.44 ± 5.80	36.60 ± 6.23	0.63
BMI [kg/m2] (Mean ± SD)	24.27 ± 5.66	24.19 ± 4.23	0.28
Smokers (%)	7.66	4.82	0.10
Ovulation day (Mean ± SD)	16.00 ± 5.08	17.96 ± 6.32	0.84
Endometrial width [mm]*** (Mean ± SD)	9.55 ± 1.99	9.57 ± 1.94	0.82

Presented as mean ± standard deviation (SD).

*Including the train and validation sets that were used to learn the model.

**At the time of embryo transfer.

***As measured on the last test day.

#### Instances creation

To train, and evaluate a real-time ovulation prediction model, multiple instances were created from each cycle in the train, validation, and test sets, where each instance included a different combination of test days from that cycle. The instances included all single test days and any combination of two test days that are at most four days apart. Training the model with additional test days or test days further apart did not improve its performance. The target class for each instance is determined by calculating the difference between the instance’s last test day and the ovulation day. The nine ovulation classes are defined as six or more days before (≤ -6), five days before (-5), four days before (-4), three days before (-3), two days before (-2), one day before (-1), the same day as (0), a day after (+ 1), or two or more days after ( ≥ + 2) the ovulation day. For example, Table 4 shows an example of a cycle with ovulation on the 13th day, while the blood tests were performed on the 3rd, 10th, 12th, and 14th days. As can be seen from the example in Table 4, seven possible instances can be generated.

**Table 4:** An example of NC-FET over the cycle days and that status at each day, where T with underline represents a test day and O with a italics represents the ovulation day. The seven possible combinations to create the instances for the presented NC-FET are presented.

Status			**T**							**T**		**T**	*O*	**T**	
Day	1	2	3	4	5	6	7	8	9	10	11	12	13	14	15

Possible combinations of days to generate the instances			
	1st Test Day	2nd Test Day	Ovulation Class
1	-	3	≤ -6
2	-	10	-3
3	10	12	-1
4	10	14	1
5	-	12	-1
6	12	14	1
7	-	14	1

For the Labeled Dataset, these instances were randomly split by the patient unique ID, into the train (60%), validation and calibration (25%), and test (15%) sets, ensuring that each patient appeared in only one set—thereby preventing any data leakage, while maintaining the same class distribution between the sets. Table 5 summarizes the dataset groups.

**Table 5:** Summary of the number of cycles and instances per dataset.

Dataset	Cycles				Instances			
	Total	Train	Validation	Test	Total	Train	Validation	Test
Labeled Dataset	3,975	2,465	967	543	21,369	14,360	4,888	2,121
Documented Ovulation	166			166	900			900
All datasets	4,141			709	22,269			3,021

The model performance results for the Labeled Dataset included 2,121 instances: a single test day conducted between the 6th and 10th cycle days, encompassing 481 instances from 391 distinct cycles, and two test days conducted on the first test day following the 6th cycle day, encompassing 1,640 instances from 509 distinct cycles.

#### Model design

Based on instances that were extracted, described in the data preparation section, a machine-learning model was trained. The model was trained on the train set to be able to classify, given an instance, the prediction of the ovulation class (≤ -6, -5, -4, -3, -2, -1, 0, + 1, ≥ +2). The features included information related to each instance consisting of baseline characteristics, alongside cycle-specific measurements derived from blood and ultrasound tests. These included both raw clinical values and calculated features reflecting hormonal and ultrasound dynamics across test days. A comprehensive description of all features is provided in Supplementary Table S1, and the extent of missing data for each feature is summarized in Supplementary Table S2. Because the model was designed to flexibly support cases both with and without ultrasound data, an appropriate handling of missing values was essential.

XGBoost classifier, a machine learning model, was selected due to the combination of its high performance and its powerful handling of missing values. Multi-class calibration using one versus rest methodology was performed as a part of the models’ development (see Supplementary Material Part 2). The grid search cross-validation method was applied and evaluated on the validation and calibration set to optimize the model hyperparameters, such as learning rate and maximal depth. After the hyperparameters optimization, the final model was trained on the training and validation sets. During the prediction stage, the ovulation class with the greatest likelihood, per instance, was selected as the model’s final prediction.

#### Natural FET treatment management algorithm (NTMA)

The objective of the Natural FET Treatment Management Algorithm (NTMA) is to autonomously manage NC-FET cycles. Embryo transfer day was calculated as the day of ovulation + plus the embryo’s developmental stage^3,4^.

This algorithm includes recommending the ideal timing for test days until an accurate prediction of ovulation can be made and a reliable suggestion for the optimal transfer day can be provided.

#### The NTMA’s objectives are as follows

- Objective 1: Transfer on the correct day according to the patient’s ovulation.
- Objective 2: Reduce the number of tests required per cycle.

#### NTMA’s logic

The algorithm’s logic considers the probability of natural ovulation to occur on each day following the last test day using the real-time ovulation prediction model. We present here a simplified logic that represents the general principles of the NTMA:

- Initial test on the 8th day of the menstrual cycle.
- Following each test day:
  - If the model predicts ovulation in 6 or more days, schedule the next test in 4 days.
  - If the model predicts that the ovulation occurred yesterday, today, or will occur tomorrow, schedule a transfer, according to the predicted ovulation day.
  - If the model determines that ovulation occurred two or more days ago (No prediction), suggest cycle cancellation, since precise ovulation day cannot be determined.
  - If neither condition is met, schedule another test one day before the predicted ovulation.

#### NTMA’s evaluation

##### Statistical evaluating approach

Measuring the performance of the NTMA without a prospective study was challenging. The algorithm’s accuracy relied on scheduling blood tests on specific days, while our dataset consisted of blood test data on days chosen by attending physicians. To address this, we use a novel statistical approach to calculate the algorithm’s performance metrics. This approach was originally proposed^21^ and factored in the likelihood of a patient ovulating on each cycle day, the prediction accuracy of the real-time ovulation day prediction model on natural cycles, and the logic of the treatment management algorithm. This was done for varying ovulation cycle days, according to the distributions observed in the data. A simplified pseudo-code for this calculation is presented in Supplementary Material Part 3. The results are presented with 95% confidence intervals, calculated using the Wilson Score method and the Monte Carlo technique. Each scenario was sampled 30 times to determine the mean, standard deviation, and confidence intervals for all evaluation metrics.

## Supplementary Information

Below is the link to the electronic supplementary material.

Supplementary Material 1

## Data Availability

Data is provided within the manuscript or supplementary information files.The data underlying this article will be shared on reasonable request to the corresponding author.

## Code availability

The code used to develop and evaluate the NTMA is publicly available at https://github.com/fertilai/nc-fet-simulator/.
