# Supplementary material for: Treatment management algorithm for natural frozen embryo transfer cycles using a real-time ovulation prediction machine learning model
Source: Sci Rep. 2026 Mar 8;16:13727. doi: 10.1038/s41598-026-42921-1 (PMC13125252; doi:10.1038/s41598-026-42921-1)

**Treatment management algorithm for natural frozen embryo transfer cycles using a real-time ovulation prediction machine learning model**

**Corresponding author**: Ettie Maman MD, *ettiemaman@gmail.com*

**Supplementary Data**

**Part 1**: **Ovulation Predictions Logic**

The ovulation day predictions of the ovulation labeling model for each treatment cycle is processed to identify the most confident prediction, by the following logic:

- If the instances’ predictions include relative-to-ovulation days -1 or 0, the instance with the highest confidence among them is selected.
- Otherwise, if the instances’ predictions include relative-to-ovulation day +1, the instance with the highest confidence in that class is chosen.
- If none of the above conditions match, the instance’s most certain prediction with the latest second test day is used.

***Part 2: Additional*** ***Data and Results***

***Table S1: Model Features***

Table S1 provides the complete list of features used in the model development.

| Patient Age |
| --- |
| Patient Height |
| Patient Weight |
| Patient Bmi |
| Test Days Distance |
| LH value on instance's 1st test day |
| E2 value on instance's 1st test day |
| P4 value on instance's 1st test day |
| E2 to P4 ratio on instance's 1st test day |
| LH value on instance's 2nd test day |
| E2 value on instance's 2 nd test day |
| P4 value on instance's 2 nd test day |
| E2 to P4 ratio on instance's 2nd test day |
| E2 change between instance's 1st and 2nd test days |
| Avg. E2 change per day between instance's 1st and 2nd test days |
| E2 on instance's 2nd to 1st test day ratio |
| P4 change between instance's 1st and 2nd test days |
| Avg. P4 change per day between instance's 1st and 2nd test days |
| P4 on instance's 2nd to 1st test day ratio |
| LH change between instance's 1st and 2nd test days |
| Avg. LH change per day between instance's 1st and 2nd test days |
| LH on instance's 2nd to 1st test day ratio |
| Follicle size on instance's 1st test day |
| N Large follicles count on instance's 1st test day |
| Endometrial thickness on instance's 1st test day |
| Follicle size on instance's 2nd test day |
| N Large follicles count on instance's 2nd test day |
| Endometrial thickness on instance's 2nd test day |
| Primary follicle size change between instance's 1st and 2nd test days |
| N large follicles count change between instance's 1st and 2nd test days |
| Endometrial thickness change between instance's 1st and 2nd test days |

***Table S2: Missing values summary***

The following table summarizes the extent of missing data for each feature across the 21,369 instances included in the Labeled Dataset.

|  | Missing Count | Missing Percent |
| --- | --- | --- |
| Patient Height | 1341 | 6.28% |
| Patient Weight | 1166 | 5.46% |
| Patient Bmi | 1345 | 6.29% |
| Follicle size on instance's 1st test day | 561 | 2.63% |
| N Large follicles count on instance's 1st test day | 561 | 2.63% |
| Endometrial thickness on instance's 1st test day | 680 | 3.18% |
| Follicle size on instance's 2nd test day | 714 | 3.34% |
| N Large follicles count on instance's 2nd test day | 714 | 3.34% |
| Endometrial thickness on instance's 2nd test day | 809 | 3.79% |

***Model Calibration***

Model calibration is essential to ensure that predicted probabilities closely mirror actual clinical outcomes, allowing for accurate interpretation. For example, if the model predicts a 70% chance for a specific ovulation day, calibration ensures that this day will actually occur around 70% of the time, making the model’s predictions more dependable in clinical practice.

Using a separate calibration set, the model achieved an expected calibration error (ECE) of 1.53 on the test set of the Labeled Dataset, significantly improving from an ECE of 5.05 without calibration. This improvement underscores the importance of calibration, making the model more reliable for supporting clinical decisions and increasing confidence in its predictions.

***Part 3: NTMA’s Performance Evaluation***

The purpose of the evaluation algorithm, initially proposed and thoroughly explained in Youngster et al. (2023), is to simulate and evaluate the performance metrics of the NTMA for managing patient cycles, without relying on a prospective clinical study. To address this, the evaluation algorithm incorporates several key components:

1. Ovulation Probabilities: We consider the likelihood of a patient ovulating on each day of the cycle (ranging from the 10th to the 21st day), based on the observed distributions in our dataset. These probabilities enable us to appropriately weight the performance metrics calculated for different ovulation days.
2. Real-Time Ovulation Day Prediction Model: The algorithm accounts for the real-time prediction model’s accuracy by considering its performance.

The performance calculation of the algorithm is carried out through three main functions.

- ***Function*** ***CalculateAveragePerformance***: This function loops over each possible ovulation day. It computes the performance of the NTMA for each option and averages the results, weighted by the ovulation probability.
- ***Function CalculateOvulationDayPerformance:*** This function calculates the ovulation day performance by iterating through the model’s predictions and probabilities. It evaluates the model’s performance using the confusion matrix (modelConfusionMatrix, see Figure 1A &1B) for the true class and then determines the appropriate action (e.g., performing tests) based on the results while using the probability to compute the performance. The actions depend on the recommendation of the NTMA, using the function TreatmentManager.Recommend
- ***Function*** ***CalculateFinalPerformance***: Once the NTMA recommends an action other than conducting further tests, this function determines the patient's final outcome (e.g., correct prediction, incorrect prediction and no prediction 7) based on the timing of the action, and the spontaneous ovulation cycle day.

Through these functions, the algorithm assesses the NTMA's overall performance, considering different options and actions based on the patient’s ovulation day and the predictions made by the ovulation prediction model. The final results are expressed with 95% confidence intervals, which were calculated using the Wilson Score method combined with Monte Carlo sampling.

**Supplementary Figure 1**: simplified pseudo-code that calculates the performance metrics of a treatment management algorithm


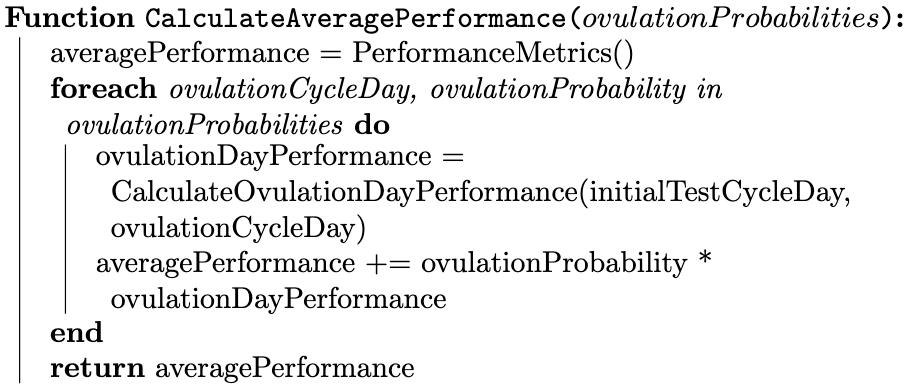


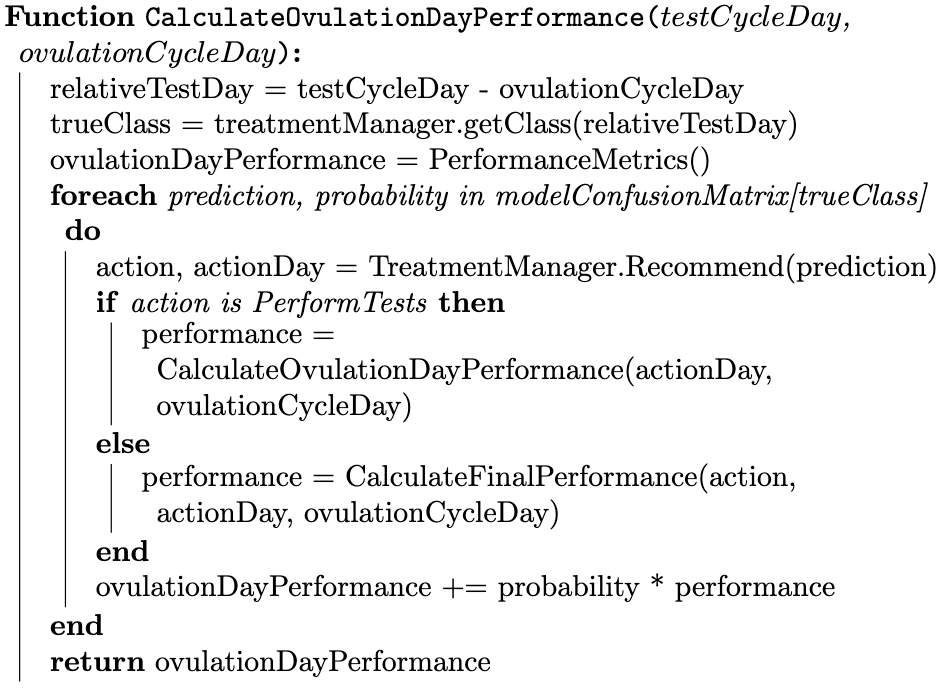


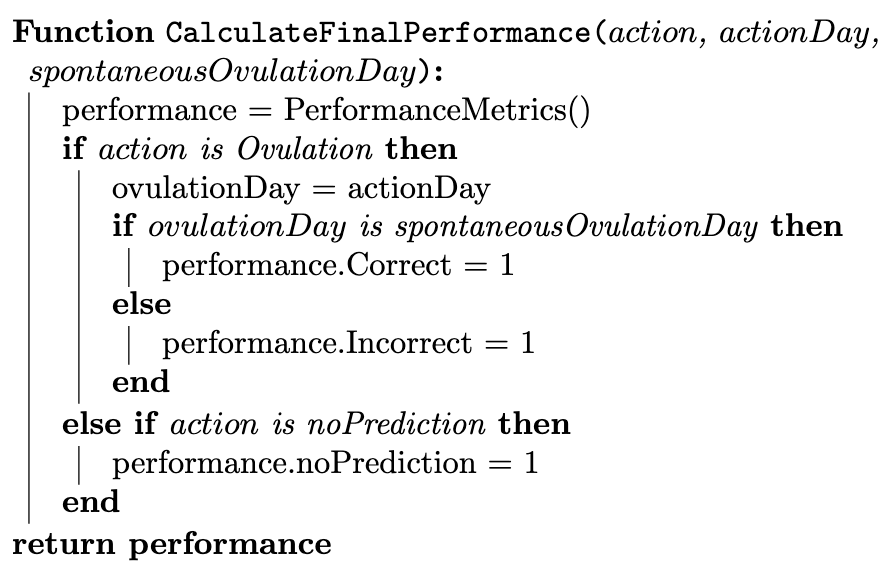

Supplement: Supplementary file 1 — Supplementary Material 1 [file 41598_2026_42921_MOESM1_ESM.docx]
